# Editorial: Neuroanatomical and molecular biomarkers for multiple sclerosis progression and therapeutic response

**DOI:** 10.3389/fnana.2025.1640594

**Published:** 2025-06-24

**Authors:** Ignacio Casanova, María Inmaculada Domínguez-Mozo, Roberto Álvarez-Lafuente

**Affiliations:** ^1^Department of Neurology, Hospital Universitario de Torrejón, Madrid, Spain; ^2^School of Medicine, Universidad Francisco de Vitoria, Madrid, Spain; ^3^Research Group in Environmental Factors of Neurodegenerative Diseases, Instituto de Investigación Sanitariadel Hospital Clínico San Carlos (IdISSC), Red de Enfermedades Inflamatorias (REI), Madrid, Spain

**Keywords:** biomarkers, multiple sclerosis, therapeutic response, progression, clinical monitoring

Multiple sclerosis (MS) is a chronic, immune-mediated disorder of the central nervous system characterized by inflammation, demyelination, and neurodegeneration. Despite substantial progress in the development of disease-modifying therapies (DMTs), the clinical course of MS remains highly variable and unpredictable, making it challenging to guide treatment and predict long-term outcomes. There is a need for biomarkers to better understand the mechanisms driving disease progression, monitor therapeutic responses, and improve personalized treatment strategies.

This Research Topic brings together four original articles that explore the landscape of neuroanatomical and molecular biomarkers in MS, focusing on the role of the immune system, neurodegeneration, and treatment response.

The critical contribution of B cells to MS pathogenesis has been underscored by the success of B-cell depleting therapies, yet their precise role in disease progression and heterogeneity remains an area of active investigation. In their comprehensive review, *Global perspectives on the contribution of b cells to multiple sclerosis: an in-depth examination and evaluation*, the authors synthesize current knowledge on B-cell subsets, antibody production, and antigen presentation, providing a global perspective on how B-cell biology informs clinical practice and therapeutic approaches (Jiang et al.).

Identifying genetic and molecular biomarkers that can predict MS susceptibility and progression is a key goal of precision medicine. In *Combining gene expression microarrays and Mendelian randomization: exploring key immune-related genes in multiple sclerosis*, the authors integrate gene expression data with Mendelian randomization techniques to uncover candidate immune-related genes associated with MS. This integrative approach highlights novel molecular targets and underscores the value of combining genetic and transcriptomic data for biomarker discovery (Ding et al.).

Multiple sclerosis (MS) is increasingly recognized as a multisystem disorder with widespread neuroanatomical alterations beyond the classic white matter lesions. In *Pineal gland volume loss in females with multiple sclerosis*, the authors investigate pineal gland morphology in MS patients, revealing a significant reduction in gland volume in female patients. These findings suggest a possible link between melatonin secretion, circadian rhythm disturbances, and MS pathogenesis (Vuković et al.).

Finally, the need for fluid biomarkers to monitor disease activity and treatment response is exemplified in *Effect of alemtuzumab over sNfL and sGFAP levels in multiple sclerosis* (Sainz-Amo et al.). Sainz-Amo et al. evaluate the impact of alemtuzumab therapy on serum neurofilament light chain (sNfL) and glial fibrillary acidic protein (sGFAP), markers of neuroaxonal, and astrocytic damage, respectively, and two of the most promising biomarkers. Their findings highlight the potential of these molecules to provide early insights into treatment efficacy and neuroprotective effects, supporting their integration into clinical practice.

Taken together, these articles emphasize the multifactorial nature of MS, where immune dysregulation, neurodegeneration, and systemic changes intertwine to drive disease progression. They highlight the promise of integrative biomarker approaches, combining neuroimaging, molecular profiling, and immunological analyses to refine disease monitoring and optimize therapeutic strategies.

Future research must focus on validating these biomarkers in diverse patient populations, standardizing methodologies for clinical implementation, and exploring how biomarker-guided approaches can improve long-term outcomes in MS.

